# A rare case of gallstone ileus—the unanswered question

**DOI:** 10.1093/jscr/rjab164

**Published:** 2021-04-29

**Authors:** Chi Fai Tsang

**Affiliations:** Department of General Surgery, Prince of Wales Hospital and Community Health Services, Sydney, New South Wales 2002, Australia

## Abstract

Gallstone ileus is caused by an impaction of one or more gallstones within the gastrointestinal tract leading to mechanical intestinal obstruction. It is a rare complication of cholelithiasis and found in 2–3% of all cases associated with recurrent episodes of cholecystitis. This case study demonstrates an atypical presentation of gallstone ileus. A 57-year-old woman was presented with abdominal pain and vomiting without previous history of gallstone disease. The features of gallstone ileus are evident on computed tomography. She underwent an emergency laparotomy and enterotomy for the removal of impacting gallstones, followed by an interval cholecystectomy and cholecystoduodenal fistula closure. This case report aims to explore the proper surgical management of gallstone ileus. Unfortunately, the question of whether interval biliary surgery should be performed remains unanswered, and surgeons will continue to make the decision based on their clinical judgement.

## INTRODUCTION

Gallstone ileus accounts for 1–4% of all presentations to hospital with mechanical small bowel obstruction. It occurs predominantly in female. In elderly patients (>65 years) it consists of 25% of all cases of small bowel obstruction [1, 2]. Gallstone ileus is caused by an impaction of one or more gallstones within the gastrointestinal tract leading to mechanical intestinal obstruction. It is a rare complication of cholelithiasis and found in 2–3% of all cases associated with recurrent episodes of cholecystitis [2]. However, it carries five times the risk of morbidity (20–57%) compared with other causes of small bowel obstruction. The mortality is ~7–18%. In addition, biliary malignancy may be the underlying cause in up to 15% of such cases [3]. Often following an episode of cholecystitis, the pressure effect, inflammation and ischemia from the offending gallstone causes the erosion through the gallbladder wall and subsequent fistula formation between the gallbladder and the adhered portion of the gastrointestinal tract [4]. Due to the proximity between the gallbladder and the duodenum, cholecystoduodenal fistula is the most common (32.5–96.5%). Less commonly, the stomach (0–13.3%), small bowel (0–2.5%) and the colon (0–10.9%) may also be involved [5]. This case report aims to explore the most acceptable operative management in the literature for gallstone ileus.

## CASE STUDY

A 57-year-old woman presented to emergency department (ED) with 3 days history of left lower quadrant abdominal pain. The pain was constant, cramping, and non-radiating.

**Figure 1:**
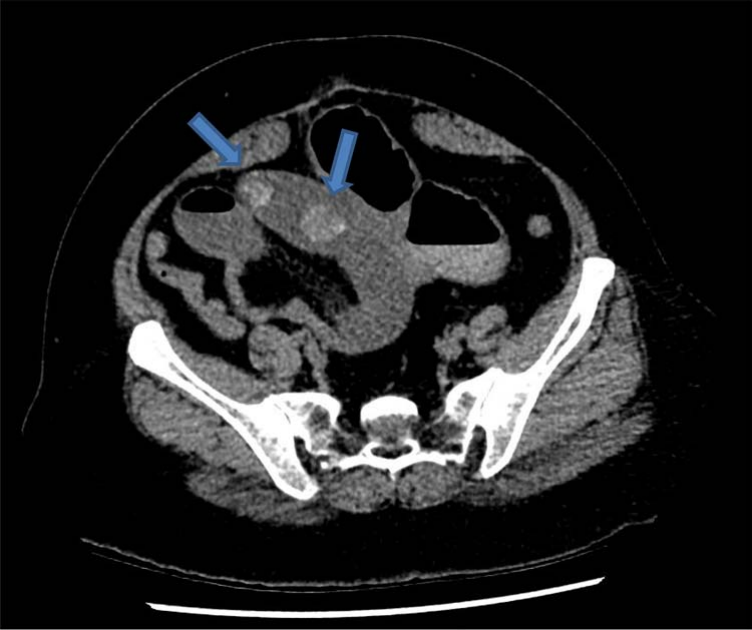
Axial CT image demonstrates two 25-mm gallstones (blue arrows) in a loop of small bowel in the right lower quadrant.

**Figure 2:**
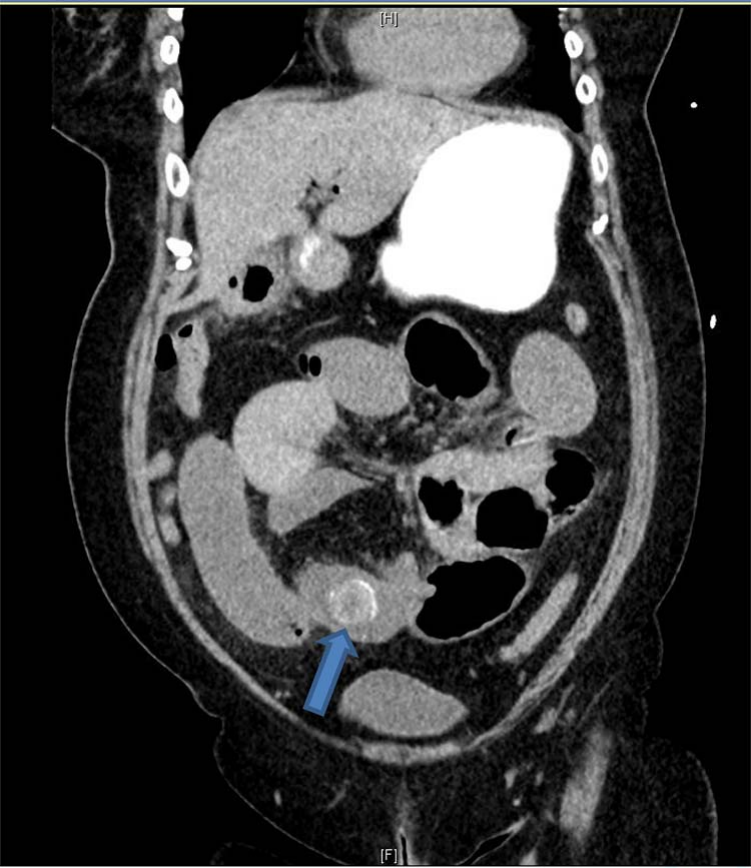
Coronal CT image demonstrates gallstones (blue arrow) (only one gallstone shown on this image) in a loop of small bowel in the right lower quadrant.

**Figure 3:**
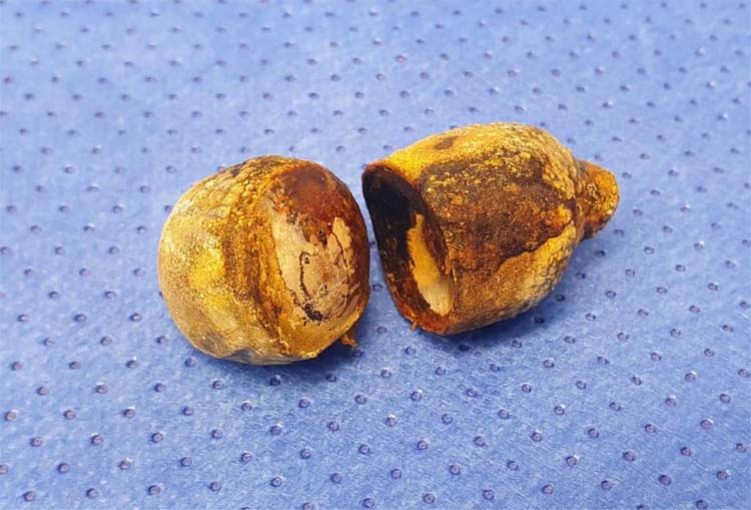
Two gallstones were delivered via enterolithotomy.

There were associated vomiting and abdominal distension. Her last bowel movement was 12 hours prior to the presentation, and she passed minimal flatus since. She has a background of chronic kidney disease. She was slightly tachycardic in ED. Abdominal examination demonstrated a mildly distended and soft abdomen with left lower quadrant tenderness without peritonism. Blood work showed an acute on chronic renal failure (creatinine 198 μmol/l and estimated glomerular filtration rate 24 ml/min/1.73 m^2^). Liver function tests were mildly deranged (bilirubin 27 μmol/l, alkaline phosphatase 105 U/l, GGT 85 U/l, alanine aminotransferase 38 U/l, aspartate aminotransferase 37 U/l) and white cell count was slightly elevated (10.7 × 10^9^/l; reference range 3.7–9.5 × 10^9^/l). Computed tomography (CT) scan (Figs 1 and 2) demonstrated multiple loops of moderated dilated small bowel. There were two 25-mm gallstones in a loop of small bowel in the right lower quadrant. The gallbladder is collapsed with intraluminal gas. There was pneumobilia in the biliary tree. The features on CT are in keeping with a gallstone ileus. Initial management included fluid resuscitation, insertion of a nasogastric tube, analgesia and nil by mouth. A Foley catheter was inserted to monitor urine output. A laparotomy proceeded within 24 hours. Two palpable gallstones were found within the distal ileum. A longitudinal enterotomy was made immediately proximal to the point of calculus impaction and two calculi were delivered from the bowel lumen (Fig. 3). There was a suspected calculus contained within the gallbladder. However, the decision was made not to proceed for further reduction via the fistula due to significant risk of duodenal injury. Her recovery was uneventful, and renal function returned to baseline. She was discharged home 7 days after her operation. During the outpatient clinic follow-up, an interval cholecystectomy and cholecystoduodenal fistula closure had been arranged in 4–6 months after her initial operation.

## DISCUSSION

Gallstone ileus is often the result of recurrent cholecystitis. In addition to the inflammation in the gallbladder, the pressure effect of the gallstone leads to erosion through the gallbladder wall and consequently causes fistula formation with the adjacent gastrointestinal tract [5]. This case study has demonstrated an atypical presentation of gallstone ileus with cholecystoduodenal fistula as the first presentation of gallstone disease. Our patient underwent an enterolithotomy and gallstone extraction in a timely fashion. Due to the residual gallstone in the gallbladder, she was then scheduled for an interval cholecystectomy and cholecystoduodenal fistula closure. While the relief of intestinal obstruction by extraction of the offending gallstone has been mostly accepted as the main therapeutic goal of surgery in gallstone ileus, the proper surgical management of this disease remains controversial [4]. The current procedures can be divided into three subgroups—enterolithotomy alone, one-stage procedure of enterolithotomy, cholecystectomy and fistula closure and two-stage procedure of enterolithotomy with an interval cholecystectomy and fistula closure [5]. In a review of 1001 cases [6], the one-stage procedure had a higher mortality rate compared with simple enterolithotomy (16.9 versus 11.7%). In the simple enterolithotomy group, 15% of patients had remaining biliary symptoms, of which only 10% required further surgeries for symptomatic relief. The recurrence for gallstone ileus was <5% in the same group. The authors concluded that simple enterolithotomy was both safe and effective. Other studies [7, 8] also supported that enterolithotomy with stone extraction alone was associated with better outcomes than more invasive techniques. Although several studies [9, 10] advocated a one-stage procedure where feasible, a review [1] suggested that it should be considered for low-risk patients only. Unfortunately, the question of whether interval biliary surgery should be performed remains unanswered, and surgeons will continue to make the decision based on their clinical judgment.

## CONFLICT OF INTEREST STATEMENT

None declared.
