# Corrigendum to: A rare case of gallstone ileus—the unanswered question

**DOI:** 10.1093/jscr/rjab250

**Published:** 2021-06-04

**Authors:** Chi Fai Tsang

**Affiliations:** Department of General Surgery, Prince of Wales Hospital and Community Health Services, Sydney, New South Wales 2002, Australia

In the original published manuscript the incorrect telephone number was listed. The manuscript has been updated with the correct telephone number, +61293822222.

